# Antibiotic prescribing in two private sector hospitals; one teaching and one non-teaching: A cross-sectional study in Ujjain, India

**DOI:** 10.1186/1471-2334-12-155

**Published:** 2012-07-12

**Authors:** Megha Sharma, Bo Eriksson, Gaetano Marrone, Suryaprakash Dhaneria, Cecilia Stålsby Lundborg

**Affiliations:** 1 Department of Pharmacology, R.D. Gardi Medical College, Ujjain, MP, 456101, India; 2 Division of Global Health, IHCAR, Department of Public Health Sciences, Karolinska Institutet, Nobels väg 9, Stockholm, Sweden; 3 Nordic School of Public Health, Box 121 33, SE 402 42, Göteborg, Sweden

## Abstract

**Background:**

The worldwide increase in antibiotic resistant bacteria is of great concern. One of the main causes is antibiotic use which is likely to be high but is poorly described in India. The aim was to analyze and compare antibiotic prescribing for inpatients, in two private sector tertiary care hospitals; one Teaching and one Non-teaching, in Ujjain, India.

**Methods:**

A cross-sectional study with manual data collection was carried out in 2008. Antibiotic prescribing was recorded for all inpatients throughout their hospital stay. Demographic profile of inpatients and prescribed antibiotics were compared. WHO Anatomical Therapeutic Chemical (ATC) classifications for antibiotics was used and Defined Daily Doses (DDD) were calculated per patient day.

**Results:**

A total of 8385 inpatients were admitted during the study period. In the Teaching hospital (TH) 82% of 3004 and in the Non-teaching hospital (NTH) 79% of 5381 patients were prescribed antibiotics. The most commonly prescribed antibiotic groups were; fluoroquinolones and aminoglycosides in the TH and, 3rd generation cephalosporins and combination of antibiotics in the NTH. Of the prescriptions, 51% in the TH and 87% in the NTH (p<0.001) were for parenteral route administration. Prescribing by trade name was higher in the NTH (96%) compared with the TH (63%, p<0.001).

**Conclusions:**

The results from both hospitals show extensive antibiotic prescribing. High use of combinations of antibiotics in the NTH might indicate pressure from pharmaceutical companies. There is a need to formulate and implement; based on local prescribing and resistance data; contextually appropriate antibiotic prescribing guidelines and a local antibiotic stewardship program.

## Background

Antibiotics are widely used medicines to treat both life threatening and trivial infections. Their indiscriminate use increases the risk of bacterial drug resistance [1,2]. High incidences of infectious diseases, high usage of antibiotics [3-5] and bacterial resistance [6] are reported from low and middle income countries. Resistant bacteria spread rapidly in these countries due to setting specific factors, such as overcrowding, poor sanitation, and a warm-humid climate. Rising rates of bacterial resistance is increasingly seen as a global problem [7-10].

Although 70% of the 1028 million people living in India live in rural areas about 80% of doctors, 75% of dispensaries and 60% of hospitals are located in urban areas [11,12]. Healthcare is provided through both public and private sector facilities. The public sector, regulated by state government, provides medical care either free or with nominal charges, and is obliged to follow national prescribing guidelines. In the private sector, patients generally pay for clinical and medical services. In India, studies on the use of antibiotics have mainly been conducted in public sector facilities, rather than private settings [13-16] where prescribing guidelines are often not implemented [17].

About 80% of the healthcare in India is provided by the private sector, and 93% of hospitals are private [12,18,19]. Hospitals are key places for antibiotic use and therefore settings for the selection and spread of resistant bacteria between patients, and finally in to the community [20-22].

This study is part of a larger project with the long term aim to formulate context relevant guidelines for the rational use of antibiotics in the study hospitals, thereby minimizing the cost of therapy and the risk of emergence of resistant organisms. The aim of this study was to analyze and compare antibiotic prescribing patterns for inpatients in two tertiary care hospitals both from private sector (one Teaching and one Non-teaching), in Ujjain district, India.

## Methods

### Setting and design

Madhya Pradesh (MP) is one of the so called *BIMARU*[23,24] states of India. *BIMARU* is an acronym of the Indian states; Bihar, Madhya Pradesh, Rajasthan and Uttar Pradesh. The term *BIMARU* resembles to a Hindi word *“Bimar”* which means ‘sick’. These states are lagging behind in economic and social development indices, as well as in healthcare performance with high infant and maternal mortality rates as compared with other states in India.

The study was conducted in the Ujjain district of MP. This has a mainly agriculture-based economy, and 61% of its 1.7 million inhabitants [11] live in rural areas. Only 23% of the villages in the district have any public medical facility [25].

The two study hospitals are both from the private sector and are tertiary care hospitals with microbiological investigational facilities. In this paper the ‘Teaching hospital’ will be referred to as TH and the ‘Non-teaching hospital’ as NTH. The TH (570 beds) was established in a rural area in the year 2005 and had inadequate transport facilities at the time of study. It is associated with a private medical college and provides free care to all patients. All the consultants in the TH receive fixed monthly salary. The management at the TH controls the purchase and supply of all medicines, which are subsequently dispensed free of charge to the patients. In the TH the generic medicines are purchased from permitted marketers.

The NTH (350 beds) was established in 1992 in the city centre and is easily accessible. Patients pay for care and no managerial control is present over prescribing practices. The prescribed medicines must be purchased by patients from pharmacies also during their hospital stay. The consultants receive extra payment for the numbers of patients they admit.

There were 11 departments in each hospital; of which 10 were comparable. The casualty inpatient department was present only in the TH and the private ward (presented as a department in this paper), only in the NTH. A local Essential Drug List was available in the TH but was not fully implemented. Local prescribing guidelines had not been formulated for either of these hospitals. In both hospitals patients are admitted from nearby villages and Ujjain city.

Hence, both the study hospitals are tertiary care hospitals from the private sector of the same district but they differ in location, type etc. The two hospitals were compared as factors, like service (free or charged), policy of payment to the prescribers, type of hospital (teaching or non teaching), location (rural or urban) etc. varied and might influence the antibiotic prescribing.

This was a cross-sectional study with data collection from April to August 2008. These are the hotter months of the year when infectious diseases such as diarrhoea are more frequent. The study included all ‘inpatients’, defined as patients who stayed for at least one night in either of the hospitals. Each time a patient was admitted to a department he/she was considered as a new patient. In this paper an inpatient that was prescribed one or more antibiotics at any stage during their hospital stay is defined as an ‘antibiotic patient’. The term ‘antibiotic’ is used for ‘anti-infectives for systemic use’ (antibacterials-J01 and anti-mycobacterials-J04), as classified by World Health Organization Collaborating Center for Drug Statistics Methodology [26] (WHOCC).

### Data collection

The data were collected manually by the nursing staff as there were no computerized prescribing records. The form used for data collection was specifically developed for the study and included patient’s name, age and sex, inpatient department number, admission and discharge dates as well as details of any antibiotics that had been prescribed (generic or trade name, dose and frequency). The form was prepared in English as the majority of the nurses were from South India and well versed in English. The nurses were trained for data collection and training was repeated for newly recruited staff. The form was pink and easily visible among the white papers of the patient file, so as to minimize the risk of missing data. One form was attached to each patient’s file on the day of admission and updated daily until final discharge or death. Thus, antibiotic prescribing was recorded for each inpatient in both hospitals during their entire hospitalization period. Computer operators were trained for data entry.

Prescribed antibiotics were classified by generic names and according to the WHO Anatomical Therapeutic Chemical Classification (ATC) [26]. Defined Daily Dose (DDD) was used as a unit to calculate the total antibiotics prescribed [26]. Some of the prescribed fixed dose combinations (FDCs) are not listed by the WHOCC, and therefore do not have ATC codes. We coded such combinations following the advice of the WHOCC and the ATC methodology up to 4^th^ level (*J01RA).

### Statistical analysis

The data was entered in Epi info (version 3.1) and Excel, and statistical analysis was completed using Stata 10.0 software (Stata Corp. College Station, Texas, USA). Frequencies and percentages were calculated for categorical variables; sum, mean, median and range for numerical variables. Chi square tests were used to compare the data between the two hospitals. The study was approved by the ethics committee of R. D. Gardi Medical College, Ujjain (approval number 41/2007).

## Results

In total, 8385 patients were admitted, 3004 in the TH and 5381 in the NTH (Table 1). Antibiotic prescribing during hospital stay was common in both hospitals, and significantly higher in the TH (82%) compared with the NTH (79%, p = 0.003). Male patients (84% and 81%) were prescribed antibiotics more often than female patients (77% and 79%) in the TH and the NTH respectively. This difference was statistically significant in both hospitals (TH-p <0.001; NTH-p = 0.003). A higher percentage of the patients in the NTH (13%) than in the TH (6% p<0.001) were in the age group 0–4 years. The highest percentage of patients prescribed antibiotics in the NTH were of 0–4 years of age. Young female patients (15–18 years) were prescribed antibiotics more often than young males, 78% and 68% respectively, in the TH. The opposite was observed in the NTH, 78% and 86%. A lower percentage of females than males, 60 years and older, were prescribed antibiotics in both hospitals.

**Table 1:** Distribution by age and sex of inpatients with percentages (in brackets) of patients that were prescribed antibiotics during their stay in a Teaching and a Non-teaching hospital in Ujjain district, India

**Age**	**Teaching hospital**			**Non-teaching hospital**		
In Years	Total	Male	Female	Total	Male	Female
0-4	179 (61)	101 (61)	78 (60)	689 (86)	403 (86)	286 (85)
5-18	337 (72)	192 (68)	145 (78)	486 (83)	324 (86)	162 (78)
19-29	533 (87)	230 (89)	303 (85)	1106 (80)	457 (85)	649 (77)
30-49	938 (84)	483 (87)	455 (82)	1465 (80)	767 (79)	698 (81)
50-59	369 (84)	204 (89)	165 (77)	480 (74)	299 (75)	181 (73)
60 and above	648 (84)	445 (89)	203 (73)	1155 (74)	713 (77)	442 (70)
Total	3004 (82)	1655 (84)	1349 (79)	5381 (79)	2963 (81)	2418 (77)

Age: As recorded at the time of admission.

A single antibiotic was prescribed to 44% and 66% of antibiotic patients and two antibiotics were prescribed to 31% and 24% antibiotic patients in the TH and the NTH, respectively. More than five antibiotics were prescribed to 3% of antibiotic patients during their hospital stay in the TH, and 0.02% in the NTH. Antibiotics were prescribed to be administered parenterally in 51% and 87% of prescribing occasions in the TH and the NTH, respectively (p<0.001). Antibiotics were prescribed for more than two weeks in 3% of antibiotic patients in the TH and in 0.2% in the NTH. A few patients (TH n = 9 out of 3004, NTH n = 22 out of 5381) were prescribed antibiotics at discharge, but not during their stay in hospital.

With respect to inter-departmental differences (Table 2), the highest percentage of patients prescribed antibiotics in the TH were in the ophthalmology department (99%), and in the NTH in the Neonatal Intensive Care Unit (NICU) (91%). The highest percentage of patients prescribed antibiotics after discharge was in the ophthalmology (83%) in the TH, and in the surgery department (55%) in the NTH. A significantly lower percentage of female antibiotic patients (26%) as compared with males (32%, p<0.001), were prescribed antibiotics after discharge in the NTH but there was no significant difference in the TH. The overall percentage of antibiotics prescribed after discharge was higher in the TH (35%) than in the NTH (29%, p<0.001). In the NICU of the NTH, 11% of patients were prescribed antibiotics after discharge, but none in the TH.

**Table 2:** Distribution of admitted patients and patients prescribed antibiotics; by department and sex in a Teaching and a Non-teaching hospital in Ujjain district, India

**Department**	**Teaching hospital**			**Non-teaching hospital**		
	**Total**	**Male**	**Female**	**Total**	**Male**	**Female**
Pediatric	229 (48) [5]	132 (45) [6]	97 (52) [3]	638 (83) [30]	453 (84) [29]	185 (82) [31]
NICU	16 (69) [0]	5 (60) [0]	11 (73) [0]	219 (91) [11]	91 (91) [13]	128 (91) [9]
Medicine	675 (71) [21]	289 (77) [18]	386 (67) [23]	1629 (73) [21]	831 (76) [22]	798 (69) [20]
Ob-Gy	216 (90) [45]	_	216 (90) [45]	417 (82) [29]	12 (75) [8]	405 (83) [30]
Surgery	695 (94) [44]	508 (94) [46]	187 (95) [38]	938 (89) [55]	639 (91) [64]	299 (86) [38]
Orthopedics	262 (72) [38]	178 (75) [34]	84 (68) [48]	266 (86) [23]	171 (88) [28]	95 (82) [13]
ENT	197 (95) [78]	111 (97) [77]	86 (92) [79]	53 (85) [38]	27 (89) [48]	26 (81) [27]
Ophthalmology	149 (99) [83]	76 (99) [80]	73 (100) [86]	7 (86) [20]	3 (100) [33]	4 (75) [75]
Pulmonary Medicine	225 (98) [41]	171 (99) [42]	54 (96) [37]	25 (88) [35]	19 (90) [16]	6 (83) [33]
ICU	116 (78) [3]	61 (77) [2]	55 (78) [6]	418 (75) [14]	256 (69) [5]	162 (75) [4]
Casualty	224 (80) [13]	124 (86) [13]	100 (75) [14]	_	_	_
Private	_	_	_	771 (74) [4]	461 (73) [32]	310 (75) [39]
Total	3004 (78) [35]	1655 (85) [36]	1349 (79) [35]	5381 (79) [29]	2963 (81) [32]	2418 (77) [26]

**n (%) [%]:** Absolute number of admitted patients (percentage of inpatients on antibiotic during hospital stay) [percentage of inpatients prescribed antibiotic at discharge]NICU = Neonatal Intensive care unit; Ob-Gy = Obstetrics and Gynecology; ENT = Ear, Nose and Throat; ICU = Intensive Care Unit.

Both median duration of hospitalization (6 versus 3 days) and median duration of antibiotic therapy (6 versus 4 days) were higher in the TH than in the NTH respectively (Table 3). DDD per antibiotic patient varied between 0.6 and 22 in the TH, and between 1 and 19 in the NTH. DDD per day generally varied between 1 and 2 in both hospitals, but for the NICU it was much less. DDDs were not specified by WHOCC for antibiotics in 9% of prescribing occasions in the TH and 3% in the NTH.

**Table 3:** Median duration of hospital stay and antibiotic treatment together with information about DDDs prescribed; by departments in a Teaching and a Non-teaching hospital in Ujjain district, India

	**Teaching hospital**							**Non-teaching hospital**					
**Department (n = number of antibiotic patients)**	**Median duration of hospital stay n (range)**	**Median duration of antibiotic treatment for antibiotic patients n (range)**	**Total DDDs used**	**DDD/antibiotic patients**	**Total days of antibiotic therapy**	**DDD/day in hospital**	**Department (n = number of antibiotic patients)**	**Median duration of hospital stay n (range)**	**Median duration of antibiotic treatment for antibiotic patients n (range)**	**Total DDDs used**	**DDD/antibiotic patients**	**Total days of antibiotic therapy**	**DDD/day in hospital**
Pediatric (n = 109)	7 (1–25)	5 (1–26)	**624**	**6**	**684**	**0.9**	Pediatric (n = 531)	**3 (1–43)**	**4 (1–44)**	**2554**	**5**	**3124**	**0.8**
NICU (n = 11)	7 (1–12)	5 (1–10)	6.3	0.6	53	0.1	NICU (n = 199)	5 (1–42)	6 (1–37)	257	1	1672	0.2
Medicine (n = 476)	4 (1–56)	4 (1–56)	3474	7	2513	1.4	Medicine (n = 1187)	3 (1–34)	3 (1–35)	10074	9	6089	1.7
Ob-Gy (n = 194)	6 (1–63)	4 (1–55)	1667	9	1179	1.4	Ob-Gy (n = 343)	4 (1–16)	4 (1–15)	2250	7	2139	1.1
Surgery (n = 654)	8 (1–88)	7 (1–89)	6911	11	5799	1.2	Surgery (n = 835)	3 (1–30)	3 (1–31)	8203	10	4577	1.8
Orthopedics (n = 188)	10 (1–69)	10 (1–63)	2963	12	2308	1.3	Orthopedics (n = 228)	5 (1–137)	5 (1–17)	1871	8	1857	1.0
ENT (n = 187)	8 (1–38)	8 (1–32)	1404	8	1653	0.8	ENT (n = 45)	3 (1–9)	4 (1–8)	339	8	194	1.7
Ophthalmology (n = 148)	5 (1–21)	7 (1–13)	904	6	883	1.0	Ophthalmology (n = 6)	3 (1–18)	2.5 (1–8)	115	19	32	3.6
Pulmonary Medicine (n = 220)	7 (1–50)	8 (1–50)	4923	22	3131	1.6	Pulmonary Medicine (n = 22)	3 (1–17)	4 (1–11)	253	12	155	1.6
ICU (n = 91)	3 (1–34)	3 (1–18)	628	7	419	1.5	ICU (n = 298)	2 (1–33)	3 (1–20)	2294	8	1261	1.8
Casualty (n = 181)	3 (1–140)	3 (1–48)	959	5	986	1.0	Casualty (n = 0)	0	0	0	0	0	0
Private (n = 0)	**-**	0	0	0	0	0	Private (n = 567)	3 (1–42)	3 (1–16)	4332	8	2856	1.5
Total	6 (1–140)	**6 (1–89)**	**24465**	**10**	**19608**	**1.2**	**Total**	**3 (1–137)**	**4 (1–62)**	**32542**	**8**	**23956**	1.4

NICU = Neonatal Intensive care unit; Ob-Gy = Obstetrics and Gynecology; ENT = Ear, Nose and Throat; ICU = Intensive Care Unit.

Trade name prescribing was more common in the NTH (96% out of a total of 412 antibiotics prescribed) than in the TH (63% of a total of 146). Greater adherence to the National List of Essential Medicines (NEDL) [27] and the WHO Model List of Essential Medicines (WHO EDL) [28] was seen in the TH (NEDL-82% and WHO EDL-72%) than in the NTH (NEDL-53%, p<0.001 and WHO EDL-35%, p<0.001). In the TH the most commonly prescribed antibiotics were ciprofloxacin (18%), metronidazole (14%), doxycycline and amikacin (7% each). In the NTH, ceftriaxone (21%), ceftriaxone with sulbactam (9%), amikacin and metronidazole (8% each) and cefoperazone with sulbactam (7%) were the most prescribed antibiotics. Oral metronidazole was more frequently prescribed in the TH (7%) than in the NTH (0.3%). A combination of ampicillin with cloxacillin was prescribed in 6% of prescribing occasions in the TH and in 1% occasions in the NTH.

The most commonly prescribed antibiotic groups (Table 4), in the TH were fluoroquinolones (23%), aminoglycosides (13%) and 3rd generation cephalosporins (12%), accounting for 48% of the total 30311 prescribing occasions. In the NTH the most commonly prescribed antibiotic groups were 3rd generation cephalosporins (31%), FDC of antibiotics (*J01RA) (25%) and fluoroquinolones (12%), accounting for 68% of the total 35534 prescribing occasions. Prescribing of anti-mycobacterials (J04) was significantly higher in the TH (8%) than in the NTH (0.2%, p < 0.001).

**Table 4:** Distribution of prescribed anti-infectives; by groups, subgroups and ATC codes; in a Teaching and a Non-teaching hospital in Ujjain district, India

**Antibiotics groups/subgroups with ATC codes**		**Teaching hospital**	**Non-teaching hospital**
		**n (%)**	**n (%)**
**Total prescribing occasions**		**30311 (100)**	**35534 (100)**
**ANTIBACTERIALS FOR SYSTEMIC USE; J01**		**25867 (85.3)**	**35427 (99.7)**
**Tetracyclines; J01A**	Tetracyclines; J01AA	2235 (7.4)	94 (0.2)
**Amphenicols; J01B**	Amphenicols; J01BA	40 (0.1)	0 (0.0)
**β- lactam antibiotics, penicillin; J01C**		3100 (10.2)	3268 (9.8)
	Penicillins with extended spectrum; J01CA	778 (2.6)	808 (2.3)
	Beta-lactamase sensitive penicillins; J01CE	3 (0.0)	18 (0.0)
	Beta-lactamase inhibitors; J01CG	5 (0.0)	2 (0.0)
	Combination of penicillin including beta lactamase antibiotics; J01CR	2314 (7.6)	2440 (7.4)
**Other beta lactam; J01D**		4333 (14.3)	11929 (33.3)
	1st generation Cephalosporins; J01DB	645 (2.1)	135 (0.4)
	2nd generation Cephalosporins; J01DC	2 (0.0)	516 (1.4)
	3rd generation Cephalosporins; J01DD	3686 (12.2)	11174 (31.3)
	Fourth-generation Cephalosporins; J01DE	0 (0.0)	51 (0.1)
	Carbapenems J01DH	0 (0.0)	53 (0.1)
**Sulfonamide with Trimethoprim; J01E**	Combination of Sulfonamide with Trimethoprim; J01EE	928 (3.1)	6 (0.01)
**Macrolides, lincosamides; J01F**		842 (2.8)	150 (0.6)
	Macrolides; J01FA	58 (0.2)	78 (0.3)
	Lincosamides; J01FF	784 (2.6)	72 (0.3)
**Aminoglycoside; J01G**		4068 (13.4)	3484 (10.2)
	Streptomycin; J01GA	212 (0.7)	4 (0.0)
	Other Aminogylcosides; J01GB	3856 (12.7)	3480 (10.2)
**Quinolones; J01M**	Fluoroquinolones; J01MA	6887 (22.7)	4156 (11.7)
Combination of antibiotics; *J01R	Combination of Antibiotics; *J01RA	1194 (3.9)	9084 (25.1)
**Other antibiotics; J01X**		2240 (7.4)	3256 (8.8)
	Glycopeptide antibacterials; J01XA	6 (0.0)	16 (0.0)
	Imidazole derivatives; J01XD	2216 (7.3)	3173 (8.6)
	Nitrofurantoin; J01XE	18 (0.1)	5 (0.0)
	Other antibacterials; J01XX	0 (0.0)	62 (0.2)
**DRUGS FOR TREATMENT OF TUBERCULOSIS; J04**		**2257 (7.5)**	**7 (0.02)**
**Drugs for treatment of Tuberculosis; J04A**			
	Antibiotics; J04AB	574 (1.9)	7 (0.02)
	Hydrazides; J04AC	570 (1.9)	0 (0.0)
	Other drugs for treatment of tuberculosis; J04AK	1113 (3.7)	0 (0.0)
**AGENTS AGAINST AMOEBIASIS & OTHER PROTOZOAL DISEASES P01;**		**2187 (7.2)**	**100 (0.3)**
**Agents against amoebiasis & protozoal diseases; P01A**			
	Nitroimidazole derivatives; P01AB	2187 (7.2)	95 (0.3)
	Other agents against amoebiasis & other protozoal diseases; P01AX	0 (0.0)	5 (0.01)

n (%): Absolute number of prescribing occasions (percentage of total prescribing occasions).

## Discussion

This is the first study which provides a detailed description of the antibiotic prescribing patterns for individual inpatients in two private sector hospitals in India. Previous studies have been age, department or disease specific; carried out in public sector settings; or in outpatient departments or commonly used comparatively less labor intensive computerized or aggregated pharmacy data [13-15,29-32]. As the private sector is a major contributor to healthcare delivery in India, this study contributes substantially to an understanding of the antibiotic prescribing patterns in the setting. A lack of computerized data makes a detailed study, like this, a time consuming and onerous exercise but at the same time leads to relatively more accurate description of the prescribing patterns.

Despite the presence of a microbiology laboratory in both hospitals, clinical samples were seldom sent for culture and sensitivity testing. Thus antibiotic prescribing in both hospitals was mainly empirical. The presence of controls in purchase and supply of medicines in the TH, seem to have encouraged generic prescribing and reduced the numbers and groups of antibiotics prescribed in the TH. In the NTH the antibiotics were mainly prescribed by trade names. Prescribing of generic medicines offers uniformity, comprehension and convenience [33,34] whereas prescribing by trade names supports a specific company and is generally not as cost effective.

In both cases, both for medicines marketed under trade names and for generic medicines, the quality of the drug at consumption is important. There is always a risk of fake or low-quality products available in the market from the producer [35,36]. This can be due to deliberate or unintentional factors during production or storage. Some antibiotics can be sensitive to, for example, hot and humid storage conditions. The presence of fake or low-quality medicines is a serious problem and stresses the importance of quality assurance during production, during the procedure of market permission and afterwards. Checking the quality of the medicines prescribed in the two hospitals was, however, not within the scope of this study.

A regular inflow of antibiotics with new trade names and newer combinations was observed throughout the study. As there is no complete list of the medicines that are available in India, finding the name of the active substance(s) for newer non-listed trade names was time consuming.

Pharmaceutical companies pursue prescribers via medical representatives (MRs) for prescribing new FDCs of antibiotics [37-39]. Parenteral prescribing and prescribing of newer and broad spectrum antibiotics were higher in the NTH than in the TH. One of the reasons might be that visits of MRs to prescribers are restricted in the TH as per policy decision, but not in the NTH. Also, regular academic deliberations and Continuing Medical Education (CME) programs held in the TH are likely to influence the prescribing there.

A total of 18 and 30 FDCs were prescribed in the TH and in the NTH respectively, out of which only 2 (co-trimoxazole, and ampicillin with clavulanic acid) are included in WHOEDL [29]. Many new FDCs of antibiotics have no underlying scientific justification. In the NTH 25% of all prescribing occasions were of new FDCs of antibiotics (*J01RA), of which almost all were a combination of 3rd generation cephalosporins with β-lactamase inhibitors. Prescribing the combinations of cephalosporin with β-lactamse inhibitor are justified in cases of ESBL producing strains of *E. coli* and *Klebsiella* and thus to keep carbapenems as reserve medicines*.* However, as most of the prescribing in our setting was empirical we cannot evaluate in what percentage of prescriptions, these combinations were justified.

In fixed dose combination of ampicillin and cloxacillin; cloxacillin is the anti-staphylococcal penicillin and is not effective against gram negative bacilli, whereas ampicillin is effective against certain gram negative bacilli but not effective against staphylococci. Staphylococcal and gram negative bacillary infections rarely coexist; except in cases of diabetic foot and some polymicrobial skin infections. In all other infections such a combination would most likely not contribute to resolve the infection. Furthermore in the available combination the dose of each drug is only half of the recommended [40]. Also both the medicines are ineffective in the case of methicillin resistant staphylococcal infections. The use of such combinations adds to the cost of therapy, results in adverse effects and encourages resistance. In light of the above facts the FDCs of ampicillin with cloxacillin and amoxicillin with cloxacillin was removed from the list of antibiotics recommended by the local Medicine and Therapeutics Committee in a Nepalese teaching hospital [41].

Despite of very few patients of diabetic foot (0.3% of total patients in the TH and 0.5% in the NTH) and polymicrobial skin infections (TH-0.5% and NTH-0.2%) admitted in the setting (unpublished data) the combination of ampicillin with cloxacillin was prescribed in 6% of prescribing occasions in the TH. The polymicrobial skin infections were mainly treated by local antibacterial medicines (Povidone-Iodine). The reason for prescribing a combination of ampicillin with cloxacillin in the setting is probably due to an empirical approach taken by the physicians in cases of unclear etiology.

Antibiotic prescribing was more common in younger patients in the NTH compared within the TH. More young female patients (5–18 years) than males were prescribed antibiotics in the TH, which may be because young female patients usually visit hospitals with more severe infections than young male patients in rural areas. This might be due to a gender bias and poorer care of girls particularly in rural areas, which has previously been noted in a healthcare utilization study in western India [29,42].

The median duration of stay and the median duration of antibiotic therapy were higher in the TH than in the NTH. One of the reasons might be the free medical care and medicines provided to the patients in the TH. However, a longer stay in hospital not only increases the risk of healthcare associated infections [43] and the use of antibiotics but also may lead to an increase the required time period of antibiotic treatment.

The results show a higher percentage of patients prescribed antibiotics in the rurally located hospital (TH), which is similar to a disease specific study done in Uttar Pradesh, India [30] but is different from a public sector study in Madhya Pradesh [44] which showed higher antibiotic prescribing rates in urban hospitals.

### Methodological considerations

The strength of this study is the detailed record of prescribing data of individual patients throughout their hospital stay. The two hospitals had many departments, so the study provides a broad view of antibiotic prescribing. The study has a novel concept, to compare the antibiotic prescribing in two private sector hospitals (TH and NTH) in same area. The data presented is the result of a carefully conducted study as presented in method section.

As per WHOCC we have coded oral metronidazole as agents against amoebiasis and other protozoal diseases (P01), and not as anti-infectives (J01). In NEDL [27], however, oral metronidazole is also listed under antibacterials for anaerobic infections, and was therefore considered as an anti-infective.

The DDD is the best available technical unit for comparing the use of medicines within and between different countries, including different drug substances [31,45,46]. However, as it is based on adult dosages, adult and pediatric patients cannot be compared. Therefore some other unit, such as prescribing occasion, as used in this paper, is also needed for comparative purposes. In addition, doses sometimes vary according to the diagnosis, but only one DDD per generic substance is given by WHOCC.

The manual data collection was possible because of the high levels of commitment of the nursing staff of both hospitals. There will however, be inevitably be some missing data.

## Conclusions

This study shows extensive antibiotic prescribing in both hospitals, and highlights major problems of empirical antibiotic prescribing. The results also point towards a situation of common prescribing of newer groups of antibiotics, and the use of trade name prescribing in both the hospitals. Lower adherence to NEDL and extensive use of broad spectrum and newer groups of antibiotics in the NTH was found. The result shows an urgent need to follow antibiotic prescribing patterns for a longer period and to formulate and implement contextually appropriate antibiotic prescribing guidelines, based on local antibiotic prescribing and resistance patterns. There is also a need of continuous local antibiotic stewardship program. The need of periodic updating of NEDL, as done for WHO EDL, was also felt.

## Competing interest

The authors declare that they have no competing interests.

## Authors’ contributions

MS, BE, SPD and CSL participated in the conception and design of the study and revising the paper critically for substantial intellectual content. MS was responsible for supervision of data collection, data analysis, training of the nursing staff, and has drafted the manuscript. GM and BE contributed to the statistical analysis. All authors have read the manuscript to revise it critically and have approved the final manuscript.

## Pre-publication history

The pre-publication history for this paper can be accessed here:

http://www.biomedcentral.com/1471-2334/12/155/prepub
